# Reduced serum IgG galactosylation is associated with increased inflammation during relapses of neuromyelitis optica spectrum disorders

**DOI:** 10.3389/fimmu.2024.1357475

**Published:** 2024-03-21

**Authors:** Shiyu Gao, Xin Jiao, Ruoyi Guo, Xiujuan Song, Bin Li, Li Guo

**Affiliations:** ^1^ Department of Neurology, The Second Hospital of Hebei Medical University, Shijiazhuang, China; ^2^ Key Laboratory of Neurology (Hebei Medical University), Ministry of Education, Shijiazhuang, China; ^3^ Key Laboratory of Neurology of Hebei Province, Shijiazhuang, China

**Keywords:** neuromyelitis optica spectrum disorders, IgG galactosylation, inflammatory demyelinating diseases of the central nervous system, neuroinflammation, IgG glycosylation

## Abstract

**Background and Objective:**

Post-translational modifications of antibodies, with a specific focus on galactosylation, have garnered increasing attention in the context of understanding the pathogenesis and therapeutic implications of autoimmune diseases. However, the comprehensive scope and the clinical significance of antibody galactosylation in the context of Neuromyelitis Optica Spectrum Disorder (NMOSD) remain enigmatic.The primary aim of this research was to discern disparities in serum IgG galactosylation levels between individuals in the acute stage of NMOSD relapse and their age- and sex-matched healthy counterparts.

**Methods:**

A total of fourteen untreated NMOSD patients experiencing an acute relapse phase, along with thirteen patients under medication, were enrolled, and an additional twelve healthy controls of the same age and gender were recruited for this investigation. Western blot and lectin enzyme techniques were used to determine the level of IgG galactosylation in the serum samples from these subjects. The expression of CD45^+^, CD3^+^, CD3^+^CD4^+^, CD3^+^CD8^+^, CD19^+^, and CD16^+^CD56^+^ in peripheral blood leukocytes was measured by flow cytometry. The enzyme-linked immunosorbent assay (ELISA) was also used to quantify the amounts of IgG. Magnetic particle luminescence assays are used to detect cytokines. Robust statistical analysis was executed to ascertain the potential associations between IgG galactosylation and the aforementioned immune indices.

**Results:**

In the context of NMOSD relapses, serum IgG galactosylation exhibited a notable decrease in untreated patients (0.2482 ± 0.0261), while it remained comparatively stable in medicated patients when contrasted with healthy controls (0.3625 ± 0.0259) (*p*=0.0159). Furthermore, a noteworthy inverse correlation between serum IgG galactosylation levels and the Expanded Disability Status Scale (EDSS) score during NMOSD relapse was observed (r=-0.4142; *p*=0.0317). Notably, IgG galactosylation displayed an inverse correlation with NMOSD relapse among peripheral blood CD45^+^, CD3^+^, CD3^+^CD8^+^, CD19^+^ cells, as well as with IL-6 and IL-8. Nevertheless, it was not determined whether IgG galactosylation and CD3^+^CD4^+^ T cells or other cytokines are statistically significantly correlated.

**Conclusion:**

Our research identified reduced IgG galactosylation in the serum of NMOSD patients during relapses, significantly correlated with disease severity, thereby providing a novel target for the diagnosis and treatment of NMOSD in the realm of medical research.

## 1 Introduction

Neuromyelitis optica spectrum disorder (NMOSD) is recognized as a rare and severe inflammation-related affliction of the central nervous system (CNS), characterized by debilitating relapses of optic neuritis and myelitis, often leading to misdiagnosis as multiple sclerosis. Since the discovery of IgG autoantibodies against aquaporin 4 (AQP4), a CNS-specific astrocyte protein, a specific antibody associated with NMOSD (1), the diagnostic process has been revolutionized. This has greatly enhanced the precision and timing of NMOSD diagnosis, thereby enabling more effective therapeutic interventions. However, the incidence of NMOSD has been increasing annually in recent years, endangering the physical and mental well-being of those affected as well as severely impairing their quality of life.

The impact of post-translational modifications, particularly glycosylation, on IgG molecules, has been widely studied. A prominent characteristic of the CH2 structural domain of the IgG is the presence of an N-linked glycan in the double-stranded state at the asparagine site N297, which is highly conserved. This fundamental structure can be expanded by incorporating terminal galactose residues (2). According to research by Wei and colleagues (3), adding galactose residues to the terminus of the IgG Fc N-glycan brought about a modification in the three-dimensional structure of Fc, boosting the affinity of Fc for C1q, thereby improving subsequent complement activation. This confirms that Fc-galactosylation enhances the ability of IgG molecules to bind to C1q and induce complement-dependent cytotoxicity.

Numerous autoimmune diseases, such as systemic lupus erythematosus, rheumatoid arthritis, psoriatic arthritis and others, have been associated with reduced serum IgG galactosylation. It is notable that antigen-specific autoantibodies often show a more pronounced change in Fc galactosylation in contrast to total serum IgG (4). Pagan and colleagues (5) have engineered soluble glycosyltransferases for converting endogenous IgG into anti-inflammatory mediators by attaching galactose moieties. These were then administered prophylactically or therapeutically in an animal model of autoimmune arthritis and nephrotoxic nephritis, leading to a significant decrease in autoimmune inflammation *in vivo*. Despite these insights, investigations into IgG galactosylation within CNS autoimmune diseases have been limited.

Previous studies have investigated the glycosylation of IgG in the cerebrospinal fluid and serum of patients with multiple sclerosis, revealing distinct glycosylation patterns (6). Glycosylation of IgG in the cerebrospinal fluid of MS patients was found to be associated with a pro-inflammatory profile (7), as evidenced by a significant reduction in IgG sialylation levels and an increase in galactosylation (8), correlating with an increased MRI lesion burden (9). Selective IgG deglycosylation using bacterial-derived endoglycosidase S (EndoS) was found to neutralise the pathogenic function of NMO-IgG in NMOSD disease. This also reduced complement-dependent cytotoxicity and antibody-dependent cell-mediated cytotoxicity (10). Moreover, EndoS function was observed to impair B-cell activity in an experimental autoimmune encephalomyelitis model (11). However, there is currently a dearth of studies aimed at quantifying IgG galactosylation levels during relapse or remission in patients with NMOSD.

The primary aim of this research was to examine changes in serum IgG galactosylation levels during the acute phase of NMOSD relapse, determining whether IgG galactosylation is affected by drug administration before and after disease onset. Moreover, this study aimed to investigate the influence of immune cells and inflammatory factors, contributing to a deeper understanding of the immune mechanisms involved in NMOSD pathogenesis. We hope that this study will identify new therapeutic strategies for managing this complex condition.

## 2 Materials and methods

### 2.1 Study participants

The study included NMOSD patients in the relapse phase who were admitted to the Neurology Department of the Second Hospital of Hebei Medical University, Shijiazhuang, China, during the period from December 2021 to November 2022. The eligibility criteria included: (1) compliance with the 2015 International Consensus Diagnostic Criteria for NMOSD (12); (2) manifestation of the initial occurrence or relapse in the acute phase; and (3) the absence of concurrent autoimmune disorders or infections. The control group was comprised of an equivalent number of healthy individuals who were age- and sex-matched and were recruited from the hospital’s physical examination center. Fasting peripheral venous blood samples of approximately 2 mL were taken from NMOSD patients before or after treatment. Spinal cord MRI data were collected from NMOSD patients in the acute phase using standardized protocols. Scans were performed with a high-resolution MRI scanner and dedicated spinal coil, covering the entire spinal cord with optimized imaging sequences including T1-weighted, T2-weighted, FLAIR, and gadolinium-enhanced T1-weighted sequences. Images were reviewed by neuroradiologists, and relevant clinical data were recorded. We recorded clinical data on these patients and divided them into two groups, defining those who received no medication at the time of blood collection as “Relapse Naive” and those who received high-dose steroid intravenous immunoglobulin or plasma exchange as “Relapse Administered”. Healthy controls provided approximately 2 mL of peripheral blood collected from non-anticoagulant tubes during routine physical examinations. This blood was employed for analysis. During the study visit, all patients underwent neurological examinations and were evaluated utilizing the Kurtzke Expanded Disability Status Scale (EDSS) (13). **Table 1** presents the demographic information of the study participants. Prior to participation, each participant gave their informed consent. The study was approved by the Ethics Committee of the the Second Hospital of Hebei Medical University (Approval No. 2021-R513) and followed the principles outlined in the Declaration of Helsinki.

**Table 1:** Epidemiological and clinical characteristics of the population in the study.

	HC	Relapse of NMOSD	
		RN	RA
Number, n	12	14	13
Age, years, mean (SD)	38 (5)	46 (13)	50 (13)
Female/male, n/n (% female)	11/1 (92)	14/0 (100)	12/1 (92)
Type of last attack, n (%)			
Optic neuritis only	n.a.	5 (36)	2 (15)
Limb paralysis	n.a.	5 (36)	3 (23)
Sensory abnormalities	n.a.	4 (28)	8 (62)
EDSS, median (IQR)	n.a.	3.75 (3-5)	8 (5-8.5)

NMOSD neuromyelitis optica spectrum disorders, HC healthy control, RN relapse naive, RA relapse administered, n number, SD standard deviation, EDSS Expanded Disability Status Scale, IQR interquartile range, n.a. not applicable.

### 2.2 Lectin-enzyme assay and enzyme-linked immunosorbent assay

After allowing whole blood samples to stand at room temperature for 1 hour, following centrifugation at 2600 rpm for 20 minutes at 2-8°C, the supernatants were removed and put into sterile Eppendorf tubes, then stored at -80°C. Microtiter plates coated with an anti-IgG capture antibody (Elabscience, China) were brought to room temperature 20 minutes prior to use. Serum samples were stepwise diluted twice to 1:2,000 for the assessment of galactosylation. For IgG quantification, the samples were stepwise diluted threefold to 1:2×10^6^. One and a half hours were spent incubating the diluted samples, 100 µl of which were added to each well of the microplate at 37°C. After discarding the liquid from the wells, a biotinylated Ricinus communis agglutinin I (Vecter Labs, USA) was added at a dilution of 1:5000 to detect IgG galactosylation. Following an additional hour of incubation at 37°C, the wells were washed three times for one minute each using the kit’s wash solution. Subsequently, streptavidin labeled with HRP was added, and incubation proceeded at 37°C for 30 minutes. Following the preceding step, the plate was washed five times, after which TMB substrate was added for color development. Absorbance values were determined in accordance with the IgG assay instructions in the kit.

### 2.3 Lectin blot

Serum samples were diluted with saline. Each sample contained 10 µg of total protein of the serum, was loaded onto a 10% sodium dodecyl sulfate-polyacrylamide gel for electrophoresis. The proteins were then moved to membranes in accordance using a standard transfer protocol. The membranes were blocked with 3% bovine serum albumin diluted with Tris-buffered saline containing Tween 20 (TBST) on a shaker at room temperature for 60 minutes. Thereafter, they were immersed in a diluted biotinylated Ricinus communis agglutinin I (1:2000) solution 4°C over night. Subsequent to rinsing the membranes with TBST, they were incubated in a diluted solution of Streptavidin labeled with Alexa Fluor 680 (Bioss, China) at a concentration of 0.1 µg/mL. The incubation lasted at room temperature, shielded from light, for 1 hour. After washing with TBST, the membranes were visualized using the Odyssey platform (LI-COR Biotechnology, USA).The intensity of each band was analyzed with ImageJ software.

### 2.4 Magnetic particle luminescence assay for serum cytokines in patients with the acute phase of NMOSD

Serum samples were collected from patients with NMOSD in the acute phase using the methods previously described. The Twelve Test Kits for Cytokines (Magnetic Particle Luminescence) (Atomlife, China) were used to perform the test strictly following the instructions. A mixture of capture microspheres from the kit containing polystyrene magnetic microspheres with different fluorescence codes was added to the reaction wells. The microspheres were then coupled with different monoclonal antibodies, including IL-1β, IL-4, IL-6, IL-8, IL-10, IL-17, TNF-α, and IFN-γ, on the surface of each microsphere. After resting and discarding the supernatant, biotin-labelled detection antibodies and serum samples were added to the reaction wells. The mixture was incubated at 37°C, protected from light, for 1 hour to form a double-antibody sandwich immune complex. The complex was then washed twice with the washing solution provided in the kit. Phycoerythrin-labelled streptavidin was added and incubated at 37°C for several minutes. After washing, the sample was tested on the Luminex MAGPIX machine, a liquid suspension microarray detector manufactured by Luminex Corporation. Samples were tested only if their results fell within the expected range, using the complex cytokine quality control products provided in the kit. The standard curve was established using the standard kit for cytokine calibration.

### 2.5 Flow cytometry detection of human lymphocyte subpopulations

For each NMOSD patient sample during the acute phase of relapse, it was essential to accurately label a BD Trucount™ Tube (BD Biosciences, USA) with the sample identification number for absolute counts to ensure proper data tracking. Subsequently, 50 µL of well-mixed, anticoagulated whole blood (using EDTA as the anticoagulant) was carefully pipetted into the bottom of the tube containing fluorescently labeled CD45, CD3, CD19, CD16, CD56, CD4, and CD8 antibodies (BD Biosciences, USA). After blood addition, the tube was securely capped and gently vortexed for 5-10 seconds to thoroughly mix the contents while minimizing cell disruption. Following this, the tube was incubated in the dark at room temperature for 15 minutes to allow optimal antibody binding. After the incubation period, 450 µL of BD FACS™ Lysing Solution (BD Biosciences, USA) was added to the tube. The tube was then recapped and gently vortexed to ensure homogeneous mixing of the lysing solution with the blood sample. The tube was incubated for an additional 10 minutes in the dark at room temperature to facilitate red blood cell lysis. Once the incubation was complete, the sample was prepared for analysis on the BD FACSCanto™ II flow cytometer (BD Biosciences, USA). After eliminating duplicate cells, each subtype of leukocytes was differentiated by plotting forward and side scatter heights, and gating was performed based on cellular morphological characteristics.

### 2.6 Cell-based assay method for AQP4-IgG titer detection in NMOSD

Serum samples collected from NMOSD acute patients were analyzed using the CBA method to determine AQP4-IgG titers. Samples were stored at -80°C until analysis. The assay involved incubating patient sera with AQP4-expressing cells, followed by detection using fluorescently labeled secondary antibodies. Titers were calculated based on fluorescence intensity measured via flow cytometry and standard curves from positive controls.

### 2.7 Statistical analysis

The OD values of lectins, which represent binding to IgG-Fc, were compared between NMOSD patients and HC patients, as were the OD values of IgG. Graphs were plotted using GraphPad Prism 9.0, and all statistical analyses were performed using the SPSS 26 statistical package (IBM, USA). Continuous variables were reported as mean ± SEM for normal distributions and as medians with interquartile ranges (IQR) for irregular distributions. The Kolmogorov-Smirnov test was used to determine the normality and homogeneity of variance of all the data. For two-group comparisons, p-values were calculated using one-way Student t-tests for data with a normal distribution and Mann-Whitney nonparametric tests for data with an irregular distribution. When there were several comparisons, the LSD method was used for *post hoc* comparisons and one-way ANOVA was used to determine p-values for continuous variables. The nonparametric Kruskal-Wallis test was used to analyze data that were not normally distributed. Significance levels were denoted as * (p < 0.05) for statistically significant two-sided p-values less than 0.05. The Pearson rank correlation and Spearman correlation tests were used to assess correlations between scores.

## 3 Results

### 3.1 Clinical and demographic characteristics of study participants

The study enrolled 27 patients in the relapse phase of NMOSD, consisting of 14 untreated individuals and 13 subjects who received high-dose hormones, intravenous human immunoglobulin, or plasma exchange, either alone or in combination. Additionally, 12 healthy controls, matched for sex and age, were included for comparison. **Table 1** provides an overview of the characteristics of all study participants.

### 3.2 Reduced levels of serum IgG galactosylation were observed in untreated patients with NMOSD relapses

**Figure 1:**
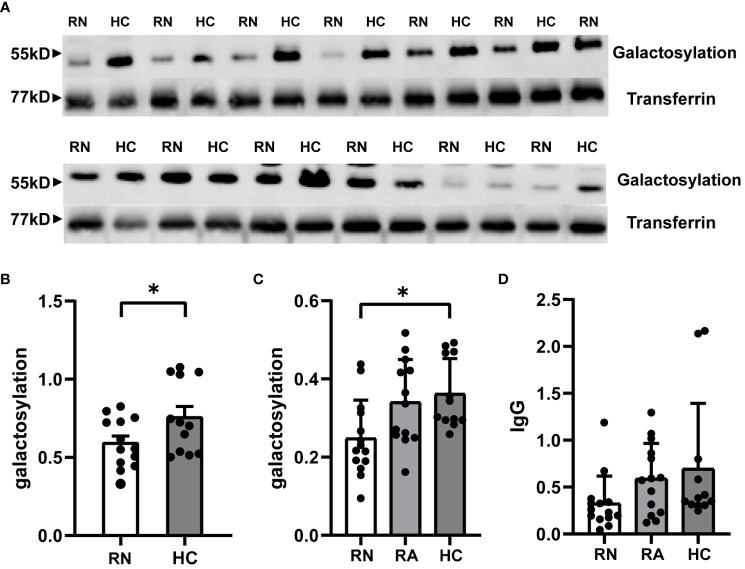
Expression of IgG galactosylation in peripheral serum of RN and RA groups in NMOSD patients and HC. Serum was extracted from patients and age- and sex-matched HC and processed for both ELISA and Western blot. IgG galactosylation of NMOSD in the RN group with HC compared in representative western blot image **(A)** and image grey scale statistics **(B)**. Each dot represents the mean of 2 or 3 independent WB and corresponding HC per patient(HC: n = 12;RN: n = 13). ELISA results quantifying the frequency of RN and RA groups in NMOSD compared to galactosylated IgG **(C)** and IgG **(D)** of HC. The data are depicted in scatter plots with histograms, presenting the median and interquartile range (or mean and standard error of the mean). Each point represents a separate subject. HC: n=12; RN: n=14; RA: n=13. HC: Healthy Control; RN: Relapse Naive; RA: Relapse Administered. *p<0.05.

ELISA results revealed that the untreated NMOSD group’s serum IgG galactosylation was considerably lower than that of Healthy Control group(Relapse Naive (RN): 0.2482 ± 0.0261, Healthy Control (HC): 0.3625 ± 0.0259; p=0.0159; n=39) (**Figure 1C**). Furthermore, the group of NMOSD patients who had received treatment showed lower levels of galactosylation compared to the untreated group, although not reaching statistical significance(Relapse Administered (RA): 0.3409 ± 0.0302; p = 0.0524). The RA group and the HC group did not, however, differ significantly. To account for variations in serum total IgG levels, we quantitatively measured total IgG in all subjects using ELISA, which showed no significant differences between the groups (**Figure 1D**). Western blot results also confirmed the significant reduction in serum IgG galactosylation in RA patients compared to HCs (**Figures 1A, B**). These findings collectively suggest that serum IgG galactosylation levels decrease during NMOSD relapse and increase after receiving high-dose hormone therapy, intravenous immunoglobulin, and plasma exchange treatment, implying a potential role of treatment in raising serum IgG galactosylation levels.

### 3.3 Serum IgG galactosylation decreases during NMOSD relapses, signifying disease exacerbation

**Figure 2:**
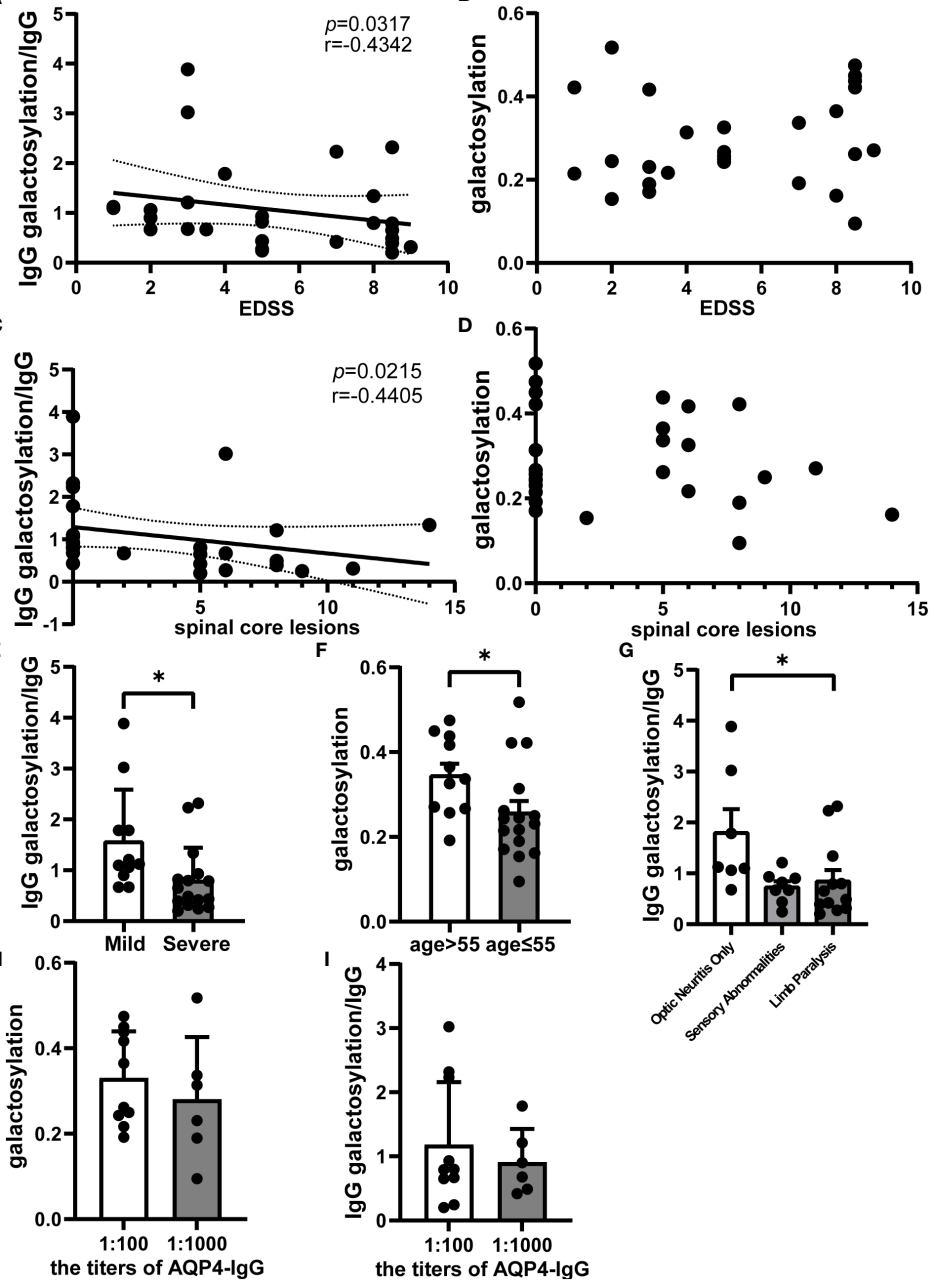
Analysis of serum IgG galactosylation and clinical parameters in NMOSD relapse. Galactosylation of IgG and IgG as measured by ELISA. Correlation analysis images of EDSS scores with **(A)** the ratio of IgG galactosylation to IgG (n=27) and **(B)** IgG galactosylation (n=27); **(C)** Spearman correlation analysis between the number of spinal cord lesion segments in NMOSD patients during acute phase attacks and the ratio of IgG galactosylation to IgG (n=27); **(D)** Scatter plot depicting the relationship between IgG galactosylation and the number of affected spinal cord segments (n=27). **(E)** Comparison of the ratio of IgG galactosylation to IgG between the Mild (EDSS ≤ 4, n=11) and Severe (EDSS>4, n=16) patient groups. The ratio is defined as the proportion of IgG galactosylated relative to each unit of IgG. **(F)** Differences between ratios of IgG galactosylation to IgG among various onset symptoms, including patients with Optic Neuritis Only (n=7), optic neuritis combined with limb paralysis (Limb Paralysis, n=12) and sensory abnormalities with or without optic neuritis (Sensory Abnormalities, n=8). **(G)** Variations in IgG galactosylation were observed between NMOSD patients aged 55 or below (n=11) and those aged over 55 (n=16). **(H)** The chart illustrates the differences in AQP4 antibody titers among NMOSD patients and their corresponding ratio of IgG galactosylation to IgG (n=16), while **(I)** provides a comparison with IgG galactosylation itself (n=16). The data are depicted in scatter plots with histograms, presenting the median and interquartile range (or mean and standard error of the mean).Each dot represents an individual subject. Dashed lines represent the range of 95% confidence intervals.*p<0.05.

Spearman correlation analysis within the NMOSD group revealed a negative correlation between the ratio of serum IgG galactosylation to IgG and the EDSS score (r = -0.4142; *p* = 0.0317; n=27) (**Figure 2A**). On the other hand, no meaningful association between IgG galactosylation and EDSS was detected (**Figure 2B**). Moreover, the ratio of serum IgG galactosylation to IgG was inversely correlated with the number of spinal cord lesion segments(r = -0.4405; *p* = 0.0215; n=27) (**Figure 2C**), while IgG galactosylation itself showed no correlation with the number of affected spinal cord segments (**Figure 2D**).These results suggest that as the EDSS score increases, indicating worsened clinical symptoms, the serum IgG galactosylation ratio decreases. Stratifying EDSS scores into mild and severe groups (EDSS ≤ 4 points), the mild group exhibited significantly higher serum IgG galactosylation ratios compared to the severe group (Mild: 1.4315 ± 0.3490, Severe: 0.8494 ± 0.1642; *p* = 0.0356; n=27) (**Figure 2E**). Analysis of specific symptoms revealed that patients with only optic neuritis attacks had significantly higher serum IgG galactosylation ratios compared to those with both optic neuritis and limb paralysis (Optic Neuritis Only: 1.8071 ± 0.4527, Limb Paralysis: 0.8519 ± 0.2118; *p* = 0.0343; n=27) (**Figure 2F**). Still, there was no statistically significant variation between the patients with sensory abnormalities with or without optic neuritis. Additionally, age was found to correlate with variations in serum IgG galactosylation (**Figure 2G**). Additionally, we conducted statistical analyses to assess the relationship between different titers of AQP4 antibodies in the serum of NMOSD acute phase patients and the ratio of IgG galactosylation to IgG, as well as the relationship between AQP4 antibody titers and IgG glycosylation itself. However, we found no statistically significant differences in these associations (n=16) (**Figures 2H-I**). These results collectively suggest that serum IgG galactosylation is associated with the severity of NMOSD relapses and tends to decrease as the condition worsens.

### 3.4 Serum IgG galactosylation decrease is accompanied by increased immune cell counts during NMOSD relapses

**Figure 3:**
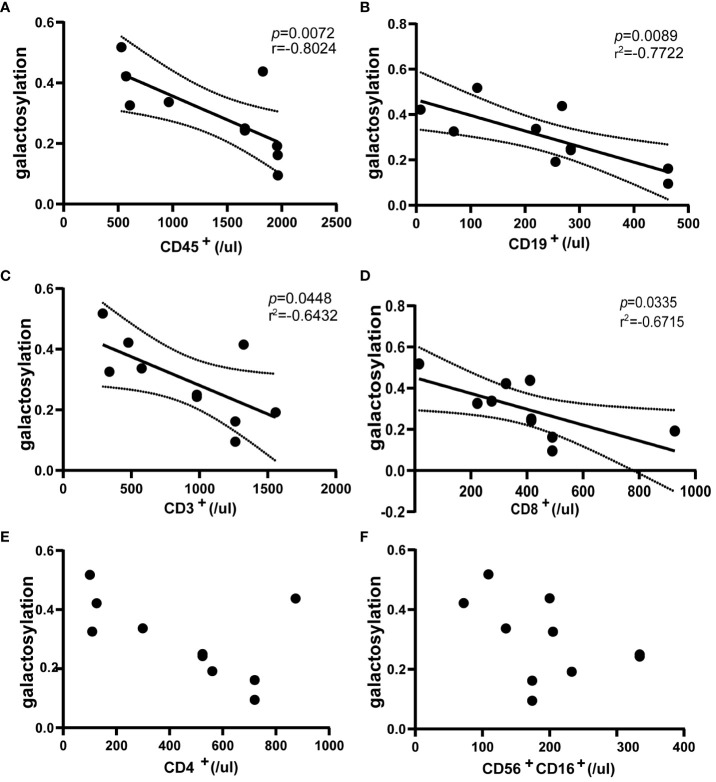
Flow cytometric assessment of the correlation between peripheral blood immune cells and IgG galactosylation in NMOSD relapses (n=10). Galactosylation of IgG as measured by ELISA, scatter plots also show the results of correlation analysis between IgG galactosylation and parameters: CD45^+^ cells **(A)**, CD19^+^ cells **(B)**, CD3^+^ cells **(C)**, CD3^+^CD8^+^ cells **(D)**, CD3^+^CD4^+^ cells **(E)**, CD56^+^CD16^+^ cells **(F)**. There was a substantial inverse correlation with IgG galactosylation and absolute counts of CD45^+^ cells **(A)**, CD19^+^ cells **(B)**, CD3^+^ cells **(C)** and CD3^+^CD8^+^ cells **(D)**. Each dot represents an individual subject. Dotted lines indicate the 95% confidence interval range. Statistically significant at a p-value less than 0.05.

The study utilized flow cytometry to analyze immune cells in the blood of NMOSD patients(n = 10) during relapse. The results demonstrated a negative correlation between serum IgG galactosylation and the absolute value of CD45^+^ cells, which represent all leukocytes (r = -0.8024; *p* = 0.0072) (**Figure 3A**). Similarly, negative correlations were observed between IgG galactosylation and absolute values of CD19^+^ B cells and CD3^+^ T cells (B cells: r = -0.7722, *p* = 0.0089; T cells: r = -0.6432, *p* = 0.0448) (**Figures 3B, C**). As IgG galactosylation decreased, the total numbers of T and B cells increased. Among T-cell subsets, CD3^+^CD8^+^ T-cell subsets exhibited a moderate negative correlation (r = -0.6715, *p* = 0.0335) (**Figure 3D**). However, absolute CD3^+^CD4^+^ T cell counts showed a moderate negative correlation with IgG galactosylation, though it did not reach statistical significance (**Figure 3E**). Regarding the absolute values of CD56^+^CD16^+^ NK cells, no discernible correlation was found (**Figure 3F**). In summary, the decrease in serum IgG galactosylation levels during NMOSD relapse was associated with an increase in the absolute counts of various immune cell subsets.

### 3.5 Reduced serum IgG galactosylation ratio correlates with inflammatory factor levels in NMOSD relapses

**Figure 4:**
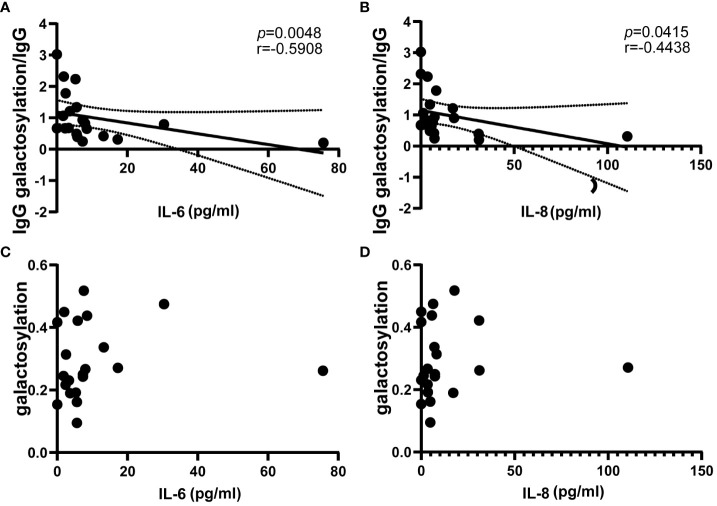
Correlation of circulating proinflammatory mediators IL-6 and IL-8 with IgG galactosylation in NMOSD relapses (n=21). ELISA was used to determine IgG galactosylation, IgG, and magnetic particle luminescence assays are used to detect IL-6 and IL-8 in serum. The ratio of IgG galactosylation to IgG was significantly negatively correlated with circulating IL-6 **(A)** and IL-8 **(B)**. There are two scatter plots on IgG galactosylation versus IL-6 **(C)** and IL-8 **(D)**, each dot represents an individual subject. Dotted lines indicate the 95% confidence interval range. Statistically significant at a p-value less than 0.05.

The study investigated the prevalence of inflammatory factors in the serum during relapse of NMOSD patients (n = 21), involving interleukins (IL), tumor necrosis factor (TNF) and interferon γ (IFN-γ). The results revealed negative correlations between the ratio of IgG galactosylation to IgG and serum levels of IL-6 and IL-8 (IL-6: r = -0.5908, *p* = 0.0048; IL-8: r = -0.4438, *p* = 0.0415) (**Figure 4A, B**). As IgG galactosylation ratio decreased, the content of IL-6 and IL-8 increased significantly. However, no significant relationship was observed between IgG galactosylation and IL (**Figures 4C, D**). Furthermore, the IgG galactosylation levels did not exhibit significant correlations with other serum inflammatory factors, namely IL-1B, IL-4, IL-10, IL-17, TNF-α, and IFN-γ (**Figures S1A–F**). These findings support the hypothesis that the decrease in serum IgG galactosylation and the increase in immune cell counts during NMOSD relapse are associated with elevated levels of specific inflammatory factors, specifically IL-6 and IL-8.

## 4 Discussion

NMOSD is now recognized as a condition characterized by astrocytosis, complement-dependent astrocyte demise, connexin dysregulation (14), and astrocyte dysfunction. These characteristics collectively represent a complex interplay of autoantibody-mediated humoral and cellular immune responses. Altered levels of total IgG galactosylation are frequently observed in various autoimmune diseases and have been associated with increased disease severity. Therefore, many researchers consider agalactosylated IgG to be pro-inflammatory. Nevertheless, there is limited research on serum IgG galactosylation in CNS inflammatory demyelinating diseases, particularly NMOSD, during both relapse and remission periods. In this study, our focus was on assessing the impact of serum IgG galactosylation on immune cells and inflammatory factors that may aid in the emergence of NMOSD. These resaults imply a potential role for IgG galactosylation in the pathogenesis of NMOSD relapse and may have implications for future treatment strategies.

Our investigation consistently observed significantly reduced serum IgG galactosylation levels during the acute phase of NMOSD relapse through quantitative and qualitative analyses, employing both Western blot and ELISA methods. These findings are consistent with previous studies. Notably, deficiencies in galactosyltransferase in lymphocytes have been associated with rheumatoid arthritis and a subsequent decrease in IgG galactosylation (15). Moreover, the activity of autoantibodies in autoimmune arthritis is influenced by galactosyltransferase activity regulation in plasma cells, leading to an increase in IgG galactosylation levels (16). Interestingly, NMOSD patients experiencing relapse and undergoing high-dose steroid treatment, intravenous immunoglobulin therapy, or plasma exchange exhibited a significant rebound in serum IgG galactosylation levels. These observations suggest that these treatments may enhance IgG glycosylation in serum and, consequently, augment the physiological anti-inflammatory capacity in NMOSD patients, in line with prior research. Following rituximab therapy, patients with immune thrombocytopenia have been shown to have elevated serum IgG galactosylation (17). In studies involving chronic inflammatory disease cohorts, not only were reductions in IgG galactosylation levels noted during the acute disease phase, but also a significant rebound was observed after treatment with azathioprine, infliximab, or vedolizumab (18). Additionally, elevated IgG galactosylation was detected in patients in remission. As a result, efforts have been made to introduce glycosylation to the FC segment of humanized IgGs’ N-glycan terminus, resulting in an augmentation of IgG’s anti-inflammatory properties (5, 19). Enhanced Fc galactosylation has significantly improved complement activation properties of monoclonal antibodies against human leukocyte antigens and human platelet antigens in patients receiving multiple platelet transfusions (20). There is some evidence that the activation threshold for C1q may be lowered due to the lower steady-state occupancy of hypogalactosylated IgG (21). This may enable pathogenic IgG to increase complement activation relative to it.

Changes in the galactosylation of both subject and pathogenic antibodies are commonly observed in autoimmune-mediated diseases. One possible explanation for this phenomenon is that these glycosylation alterations influence the binding to FcγR and C1q, as well as their downstream activation. The mechanism through which Fc galactosylation contributes to NMOSD disease may include: (i)The Fc structural domains of glycosylated IgG contribute to elevating the activation threshold of immune complexes by fostering inhibitory effects on Fcγ receptor IIB (22), thus endowing them with anti-inflammatory properties. (ii) Terminal galactosylation of Fc glycans alters the Fc’s three-dimensional conformation and promotes the hexamerization of IgG1, thereby enhancing the affinity of C1q and the subsequent activation of complement (3, 23).

Our data revealed a significant negative relationship between serum IgG galactosylation and the disease severity score EDSS during NMOSD relapse. These findings support previous research indicating a significant association between total IgG galactosylation and rheumatoid factor in the sera of rheumatoid arthritis patients (24). ACPA-IgG galactosylation levels were negatively correlated with inflammation-related measurements, including erythrocyte sedimentation rate, C-reactive protein, and rheumatoid factor (25). A cohort study on systemic lupus erythematosus conducted by Jing Han et al. revealed an increase in the extent of galactosylation of anti-dsDNA IgG in response to disease activity, as indicated by the systemic lupus erythematosus disease activity index scores (26). Moreover, anti-dsDNA IgG with a higher degree of galactosylation exhibited better performance in response to disease activity compared to total IgG. In CNS studies, IgG galactosylation in myelin oligodendrocyte glycoprotein antibody-associated disease (MOGAD) has also been found to correlate with disease activity (27). Michael J. Kemna and colleagues (4) conducted a study on anti-neutrophil cytoplasmic antibodies (ANCA) associated vasculitis, showing a reduction in both serum total IgG and antigen-specific antibody PR3-ANCA during vasculitis relapse. Serum IgG galactosylation not only reflects disease severity but also predicts the response of autoimmune diseases to immunosuppressive drugs, as demonstrated in rheumatoid arthritis by Susanna L. Lundström (28). Overall, these findings underscore the potential of serum IgG galactosylation levels as a valuable biomarker for assessing NMOSD disease activity and aiding in clinical diagnosis, highlighting the significance of glycosylation alterations in autoimmune diseases.

Our results revealed that reduced serum IgG galactosylation during NMOSD relapse was negatively correlated with the proliferation of various immune cells, with significant differences observed in CD45^+^ leukocytes, CD3^+^ T cells, CD3^+^CD8^+^ T cells, and CD19^+^ B cells. In contrast, no significant correlation was observed for CD3^+^CD4^+^ T cells and CD16^+^CD56^+^ NK cells. Some researchers have attempted to explore the relationship between IgG galactosylation and T cells and B cells, but no consensus has been reached on the mechanism. Yannic C. Bartsch et al. found that *in vitro* glycosylation of IgG autoantibodies reduced IL-6 production by dendritic cells and Th17 cell accumulation in an autoimmune model, while glycosylated IgG immune complexes increased the frequency of antigen-specific Foxp3^+^ Treg cells (29). Genome-wide studies of IgG glycosylation patterns have shown that Genes distributed throughout the immune system, particularly in B cells and antibody-producing cells, are extremely rich for IgG N-glycosylation regions (30). Furthermore, in addition to the gene encoding the beta-1,4-glycosyltransferase 1, a novel locus on chromosome 1, the Runt-related transcription factor 3 site, has a strong effect on the pattern of IgG glycosylation and is associated with galactosylation reduction (31). Animal studies have demonstrated that ungalactosylated IgG antibodies induce a proinflammatory process through a mix of T cell-dependent (TD) protein antigen and proinflammatory mutual stimulation. Ungalactosylated IgG is produced by the TD aggressive Th1 and Th17 immune responses, and the triggering of B cells and disease-causing immune responses might be inhibited by transferring a tiny quantity of specific to the antigen sialylated IgG antibody (32). Considering the significant differences between our data and the studies mentioned above, it is reasonable to assume a close relationship between IgG galactosylation and lymphocytes in the pathogenesis of NMOSD, warranting further investigation.

The findings of this investigation demonstrated a statistically significant inverse relationship between the serum IgG galactosylation ratio and IL-6 and IL-8 levels in NMOSD relapse. However, despite analyzing common inflammatory cytokines such as IL-1B, IL-4, IL-10, IL-17, TNF-α, and IFN-γ, no significant variations were observed in their levels. The immune microenvironment, which comprises various cytokines, can differentially affect IgG galactosylation in antibody-secreting cells. For instance, IFN-γ stimulation was found to increase IgG galactosylation and upregulate beta-1,4-galactosyltransferase 1 (33). *In vitro* intervention cell assays of serum from rheumatoid arthritis patients demonstrated a negative correlation between IgG of galactose-free glycoforms and TNF-α production (25). However, reports on cytokines and IgG glycosylation in NMOSD and CNS autoimmune diseases are scarce. Therefore, definitive conclusions regarding the correlation between serum IgG galactosylation and the secretion and activation of cytokines such as IL-1B, IL-4, IL-10, IL-17, TNF-α, and IFN-γ in NMOSD relapse cannot be made at this time. Future studies could explore this question in greater depth by increasing the number of cases or examining the glycosylation profile of AQP4-specific antibodies in NMOSD serum. Differences among these diseases may be attributed to different activation patterns in distinct disease contexts and the intricate interconnectedness of glycan networks that regulate in the microenvironment, which regulates the expression of glycosyltransferases, influencing the physiological roles of functional components in various cellular models.

The study provides promising evidence of serum IgG galactosylation in NMOSD relapses, adding to existing research on IgG galactosylation in autoimmune diseases and contributing to the knowledge of central nervous system autoimmune inflammatory demyelinating diseases in systemic disease studies. Prior research on blood and cerebrospinal fluid from individuals with MS revealed an altered IgG N-glycan profile, with serum levels of IgG galactosylation in MS patients considerably higher than in controls without inflammatory disease (6–8). Moreover, the pattern of galactosylation in the cerebrospinal fluid of MS patients was not significantly different from that in the blood. While our findings in NMOSD are in line with numerous autoimmune disease studies, they diverge from observations in MS due to several factors. There are several factors could account for this disparity: (a) MS is primarily an autoimmune disorder mediated by the cellular immune system where T cells, such as Th17 (34–36) and Treg (37–39), contribute significantly to its pathogenesis, and the minor pro-inflammatory effect of IgG galactosylation may be counteracted. In contrast, the pathological process of NMOSD is more linked to humoral immunity and antibody-producing cells, particularly B lymphocytes, due to the crucial role of anti-AQP4 autoantibodies in triggering the disease. Furthermore, the process of IgG galactosylation takes place in B cells and is regulated by various cytokines. (b) In the early stage of NMOSD, the cerebrospinal fluid cytokine profile exhibits upregulated IL-17 (40) and other cytokines similar to those seen in MOGAD but distinctly different from those seen in MS (41). (c) The rarity of both MS and NMOSD presents challenges in sample collection, potentially impacting the generalizability of study findings and introducing selection bias. The available studies have limited case numbers, varying geographical factors, and a lack of large-sample multicenter cohort studies, which may introduce selection bias. Monitoring the galactosylation status of total or disease-specific antibodies in NMOSD could aid in anticipating or identifying potential NMOSD occurrences, enabling intervention before or immediately after symptom manifestation.

To conclude, the observed reduction in serum IgG galactosylation during NMOSD relapse is closely associated with disease severity. Moreover, the concomitant increase in lymphocyte counts and elevation of pro-inflammatory factors provide additional insights into the pathophysiology of NMOSD relapse and may inform therapeutic strategies. Taken together, these findings underscore the potential of IgG galactosylation as a promising biomarker for monitoring NMOSD relapse severity and guiding therapeutic interventions towards personalized treatment approaches.

## Data availability statement

The original contributions presented in the study are included in the article/supplementary material, further inquiries can be directed to the corresponding authors.

## Ethics statement

The studies involving humans were approved by Ethics Committee of the Second Hospital of Hebei Medical University. The studies were conducted in accordance with the local legislation and institutional requirements. The participants provided their written informed consent to participate in this study.

## Author contributions

SG: Writing – review & editing, Writing – original draft, Visualization, Investigation, Data curation. XJ: Writing – review & editing, Writing – original draft, Resources, Methodology, Investigation. RG: Writing – review & editing, Visualization, Software, Resources, Investigation, Data curation. XS: Writing – review & editing, Resources, Methodology, Investigation, Conceptualization. LG: Writing – review & editing, Writing – original draft, Validation, Resources, Methodology, Investigation, Funding acquisition, Formal Analysis. BL: Writing – review & editing, Writing – original draft, Software, Project administration, Methodology, Investigation, Funding acquisition, Data curation.
