# Supplementary figures and images for: Reduced serum IgG galactosylation is associated with increased inflammation during relapses of neuromyelitis optica spectrum disorders
Source: Front Immunol. 2024 Mar 21;15:1357475. doi: 10.3389/fimmu.2024.1357475 (PMC10991735; doi:10.3389/fimmu.2024.1357475)

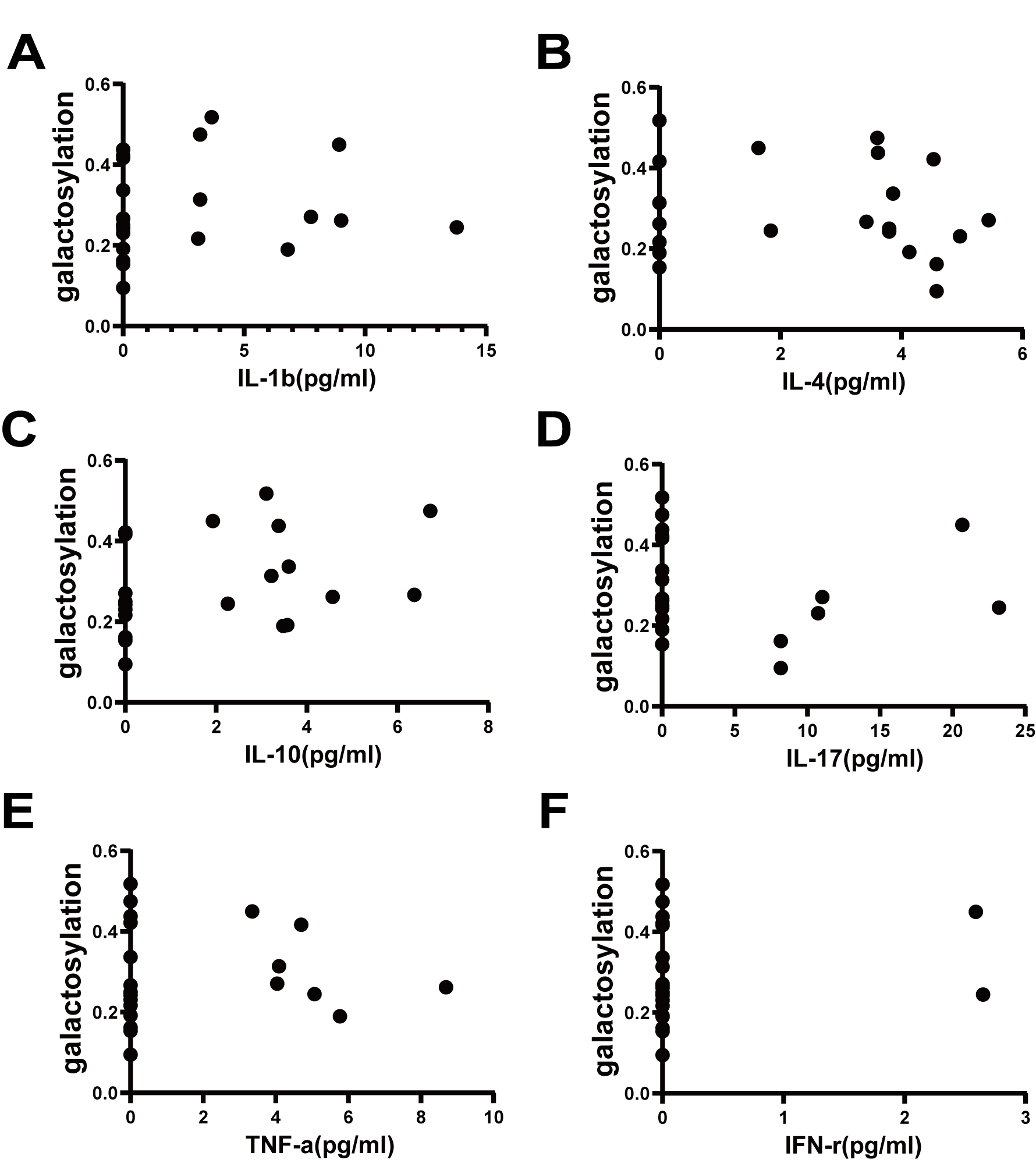

Supplement: Supplementary file 1 [file Image_1.tif]
